# Niclosamide from an anthelmintic drug to a promising adjuvant therapy for diabetic kidney disease: randomized clinical trial

**DOI:** 10.1186/s13098-023-00995-1

**Published:** 2023-02-16

**Authors:** Basma Mahrous El-fatatry, Sahar Mohamed El-Haggar, Osama Mohamed Ibrahim, Khaled Hamed Shalaby

**Affiliations:** 1 Department of Clinical Pharmacy, Faculty of Pharmacy, Tanta University, El-Guiesh Street, Tanta, 31527 Egypt; 2 Department of Internal Medicine, Faculty of Medicine, Tanta University, Tanta, Egypt

**Keywords:** Niclosamide, Diabetic kidney disease, Wnt/β-catenin, Albuminuria

## Abstract

**Background:**

Diabetic kidney disease (DKD) is a serious complication that begins with albuminuria and often leads to a rapid progressive decline in renal function. Niclosamide is a potent inhibitor of the Wnt/β-catenin pathway, which controls the expression of multiple genes of the renin–angiotensin–aldosterone system (RAAS), which in turn is influences the progression of DKD. This study was conducted to evaluate the effect of niclosamide as adjuvant therapy on DKD.

**Methods:**

Out of 127 patients screened for eligibility, 60 patients completed the study. After randomization, 30 patients in the niclosamide arm received ramipril plus niclosamide, and 30 patients in the control arm received ramipril only for 6 months. The primary outcomes were the changes in urinary albumin to creatinine ratio (UACR), serum creatinine, and estimated glomerular filtration rate (eGFR). The secondary outcomes were measurements of urinary matrix metalloproteinase-7 (MMP-7), 8-hydroxy-2ʹ-deoxyguanosine (8-OHdG), and podocalyxin (PCX). Comparisons between the two arms were done using student t-test. Correlation analysis was done using Pearson correlation.

**Results:**

Niclosamide decreased UACR by 24% (95% CI − 30 to − 18.3%) while there was a rise in UACR in the control arm by 11% (95% CI 4 to 18.2%) after 6 months (P < 0.001). Moreover, a significant reduction in MMP-7 and PCX was noticed in the niclosamide arm. Regression analysis revealed a strong association between MMP-7, which is a noninvasive biomarker predicting the activity of the Wnt/β-catenin signaling, and UACR. A 1 mg/dL decline in MMP-7 level was associated with a 25 mg/g lowering in UACR (B = 24.95, P < 0.001).

**Conclusion:**

The addition of niclosamide to patients with diabetic kidney disease receiving an angiotensin-converting enzyme inhibitor significantly reduces albumin excretion. Further larger-scale trials are needed to confirm our results.

*Trial registration:* The study was prospectively registered on clinicaltrial.gov on March 23, 2020, with identification code NCT04317430.

## Introduction

Diabetic kidney disease (DKD) develops in approximately 40% of patients with type 2 diabetes and is associated with significant mortality [1]. Diabetic kidney disease is defined clinically by persistent elevated urinary albumin creatinine ratio (UACR) > 30 mg/g, a persistent reduction in estimated glomerular filtration rate (eGFR) < 60 ml/min/1.73 m^2^, or both [2]. Albuminuria is considered the biggest predictor of kidney function deterioration and DKD progression [3].

The mechanisms behind the pathophysiology of DKD are complex and not fully understood [4]. The activated renin–angiotensin–aldosterone system (RAAS) seems to play a vital role in the progression of diabetic kidney complications. Both metabolic and hemodynamic alterations interact synergistically to activate RAAS. [5]. The use of RAAS blockers, in addition to good glycemic, and blood pressure control has been a mainstay of treatment to slow DKD progression [6]. Despite using RAAS blockers and even trials of several new agents, a relatively large number of patients are not adequately treated, and albuminuria remains and progresses to end-stage renal disease (ESRD) [7].

Recently, it was found that multiple genes of RAAS are direct downstream targets of Wnt/β-catenin. In adult healthy kidneys, Wnt/β-catenin signaling is silenced [8]. However, reactivation of Wnt/β-catenin occurs in a wide variety of kidney diseases including DKD [9]. Upon activation, the Wnt ligand binds to its receptor leading to disturbance of phosphorylation and degradation of β-catenin. Consequently, stabilization and nuclear translocation of β-catenin activate transcription factors which in turn activate promoter regions of all RAAS genes; angiotensinogen, renin, angiotensin-converting enzyme and the angiotensin II type 1 and type 2 receptors [8]. Since Wnt/β-catenin directly controls the expression of multiple RAAS genes, blockade of this signaling, in theory, could simultaneously limit RAAS activation and have a beneficial effect in diabetic kidney patients [8].

Blocking of RAAS activation by Wnt/β-catenin inhibition avoids the disadvantage of standard RAAS blockers, as the use of angiotensin-converting enzyme inhibitors (ACEIs) and angiotensin receptor blockers (ARBs) leads to compensatory upregulation of other RAAS components such as renin and leads to declined responsiveness in long-term [8]. Moreover, the activation of renal Wnt/β-catenin signaling was reported to induce podocyte injury and upregulate inflammatory pathways which could be inhibited by blocking this signal [10].

Niclosamide, which is an antihelminthic drug approved by Food and Drug Administration (FDA) since 1982, has recently demonstrated activity in many disease models, ranging from cancer and metabolic diseases to multiple types of infections through various mechanisms [11]. Niclosamide has been reported to be a potent inhibitor of Wnt/β-catenin [12]. In addition, preclinical studies showed that niclosamide could improve diabetes and DKD through inhibition of the mammalian target of rapamycin (mTOR) signaling pathway that is implicated in diabetes and DKD progression [13, 14]. Furthermore, niclosamide restores podocyte dysfunction, reduces urinary albumin, and ameliorates glomerular hypertrophy [13, 14].

Matrix metalloproteinase-7 (MMP-7), also known as matrilysin, is a secreted, zinc- and calcium-dependent endopeptidase that degrades a broad range of extracellular matrix substrates [15]. MMP-7 expression is upregulated in diseased kidneys and its expression is controlled by the Wnt/β-catenin signaling [16]. Therefore, urinary MMP-7 was measured in this study as a noninvasive biomarker that can predict the activity of the Wnt/β-catenin signaling in diseased kidneys [15]. Several studies have reported it as a predictive, noninvasive early biomarker for kidney diseases [16–18].

Podocalyxin (PCX) is a podocyte-specific protein that was reported to be an early marker for podocyte injury and diabetic nephropathy [19]. It was measured in this study to evaluate the effect of niclosamide on podocyte injury in DKD. Oxidative stress contributes to the pathogenesis and progression of DKD [20]. Urinary 8-hydroxy-2ʹ-deoxyguanosine (8-OHdG), which is a sensitive biomarker for DNA damage, has been reported to increase in patients with diabetes with micro- and macroalbuminuria compared to normoalbuminuric patients [21]. Therefore, urinary 8-OHd was measured in this study.

Both ACEIs and ARBs reported to have almost the same efficacy in DKD [22]. This clinical study was designed to investigate the effect of niclosamide on albuminuria in patients with diabetes receiving ACEIs. To the best of our knowledge, this is the first clinical study to investigate the role of niclosamide in DKD.

## Patients and methods

### Study design

This study was an open-labeled randomized controlled parallel clinical study designed to investigate the role of niclosamide in DKD. Eligible patients were randomly assigned to the niclosamide arm or the control arm. Randomization was carried out based on the days of the hospital visit every week. Patients in the niclosamide arm received ramipril plus niclosamide 1 g once daily, and patients in the control arm received ramipril only for 6 months. Ramipril was chosen as it is routinely used as standard therapy for diabetic patients with albuminuria in Internal Medicine Department, Tanta University Hospital, Tanta, Egypt.

### Patients

Patients were recruited from Internal Medicine Department, Tanta University Hospital, Tanta, Egypt. Eligible patients were aged ≥ 18 years with a confirmed diagnosis of type 2 diabetes mellitus at least 6 months before screening and had stage 2 or 3 diabetic nephropathies (persistent micro- or macroalbuminuria UACR > 30 mg/g) despite treatment with a maximum tolerated dose of ramipril for at least eight weeks before recruitment. Exclusion criteria were Type 1 diabetes mellitus, severe renal impairment (eGFR < 30 mL/min/1.73 m^2^), pregnancy or lactation, chronic heart failure, malignancy, inflammatory or autoimmune disease, and history of kidney disease other than diabetic nephropathy.

### Assessment and monitoring

At the screening visit, age, gender, height, weight, body mass index (BMI), diabetes duration, and blood pressure data were recorded. Urine samples were collected at baseline and after 6 months to assess UACR, MMP-7, PCX, and 8-OHdG using enzyme-linked immunosorbent assay (ELISA) kits. Blood samples were also collected at baseline and after 6 months to assess fasting blood glucose, glycosylated hemoglobin (Hemoglobin A1c), and serum creatinine using standard colorimetric methods.

The estimated glomerular filtration rate (eGFR) was calculated using the Chronic Kidney Disease Epidemiological Collaboration (CKD-EPI) equation as it is more accurate at higher levels of renal function and better to be used for clinical assessment of DKD [23]. The eGFR was calculated at baseline and the end of the study. The primary outcomes were the changes in UACR, serum creatinine, and eGFR after 6 months. Changes in other measured biomarkers were considered secondary outcomes.

Patients had regular visits every month for medication refills and to report if there are any encountered side effects. As far as we know, niclosamide can be taken with or without food and there is no evidence that food affects niclosamide effect. To prevent GIT upset, we advised patients to take niclosamide after a light meal. Every month, all administered medications by each patient were reviewed to exclude any patients taking medications that induce albuminuria or interact with niclosamide.

### Statistical analysis

Statistical analysis was carried out using SPSS statistical package version 28.0, May 2021, IBM corporation software group, USA. A chi-square test was applied to compare categorical clinical variables between groups. The Shapiro–Wilk test was applied to the measured parameters before running parametric statistical analysis. Normality test results reveal the normal distribution of our data with a P-value greater than 0.05. Analysis of baseline characteristics and biomarkers was done using unpaired student t-test for parametric data. Comparing the two groups mean values and the percent change in variables were done using a student t-test with statistical significance being set at P < 0.05. Correlation analysis was done using Pearson correlation. Correlation coefficients are interpreted as weak (< 0.4); moderate (0.4–< 0.7), or strong relationship (> 0.9). [24]. A linear regression test was also conducted to evaluate the association between the measured biomarkers and UACR.

Considering that the primary objective of this trial was to compare UACR between the niclosamide arm and control arm, we calculated the minimum number of patients needed to detect the 20% change in UACR. The lower limit of 20% UACR reduction was chosen as a cut-off point representing clinical relevance which is not likely to be subjected to variance error. The assumed mean of the control group was 300 mg/g, and the expected mean difference between the control and treatment groups was 60 mg/g. Assuming 80% power, a two-sided type I error rate of 0.05, an allocation ratio (r = 1), and a standard deviation equal to 75 mg/g. After applying a 15% dropout rate, 29 participants were needed in each arm of the trial. Therefore, it was planned to enroll 30 patients per arm for 1:1 randomization.

## Results

### Patient characteristics

**Fig. 1:**
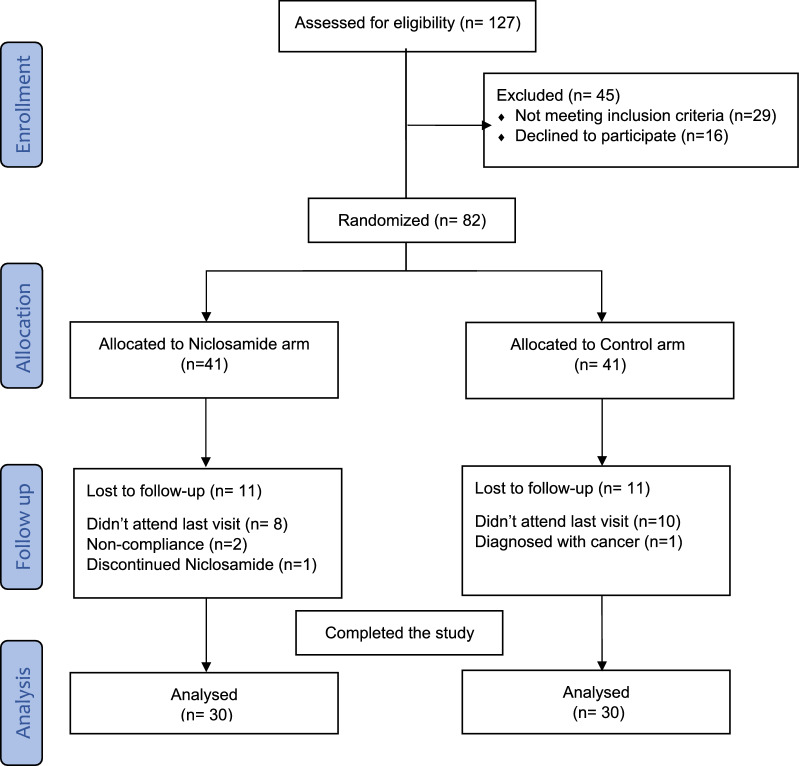
Flow chart of study participants

From March 2020 to April 2022, a total of 127 patients were assessed for eligibility. As shown in the flow diagram (Fig. 1), 82 patients were enrolled in the study and randomly assigned to the niclosamide or control arm. Eleven patients in each arm failed to return to the last outcome measure. Eventually, 60 patients completed the study and were included in the per-protocol analysis. All patients were on ramipril 10 mg at least eight weeks before recruitment and continue on it throughout the entire study duration. All patients were on regular insulin and/or oral hypoglycemic agents to control their diabetes. No major changes have been made to their diabetic regimen throughout the study duration. Baseline demographic and clinical characteristics were generally balanced in both arms as shown in Table 1.

**Table 1:** Baseline patient characteristics

Characteristics	Niclosamide arm (n = 30)	Control arm (n = 30)	P value between arms
Gender, male, No. (%)	11 (36.7%)	12 (40%)	0.795
Age, mean ± SD, years	52.7 ± 5.2	54.2 ± 5.8	0.301
Body mass index, mean ± SD	28.7 ± 3.5	29.2 ± 3.9	0.564
Obese patients, No. (%)	10 (33.3%)	12 (40%)	0.54
Hypertensive patients, No. (%)	15 (50%)	13 (43%)	0.605
Diabetes duration, mean ± SD, years	9.5 ± 2	9.7 ± 3.2	0.739
Use of insulin, No. (%)	23 (76.7%)	25 (83.3%)	0.519
Fasting blood glucose, mean ± SD, mg/mL	163.3 ± 23.2	164.9 ± 26.1	0.803
Hemoglobin A1C, mean ± SD, %	9.7 ± 1.1	9.4 ± 1	0.254
Systolic blood pressure, mean ± SD, mm Hg	127.9 ± 10.4	133 ± 11.8	0.083
Diastolic blood pressure, mean ± SD, mm Hg	81.4 ± 7.3	88.2 ± 20	0.085

Values are expressed as mean ± standard deviation or number (percent)

### Primary outcomes

As shown in Table 2, administration of oral niclosamide 1 g daily for 6 months, in addition to a routinely used ACEI, was associated with a 24.2% decrease in baseline albumin to creatinine excretion (95% CI, − 30 to − 18.3%), in contrast, to control arm which showed an 11.1% increase in baseline UACR (95% CI, 4 to 18.2%). The Niclosamide arm showed a non-significant change in serum creatinine and eGFR after 6 months compared to baseline. On the other hand, a significant increase in serum creatinine and a significant reduction in eGFR was revealed in the control arm after 6 months compared to baseline. Comparing the percent change in all primary outcomes (including UACR, creatinine, and eGFR) between the two arms, a statistically significant difference was revealed with superiority to the niclosamide arm (P < 0.05).

**Table 2:** Summary of the primary outcomes

	Niclosamide arm n = 30	Control arm n = 30	P value between arms
UACR (mg/g)			
Baseline	225.5 ± 83	247.7 ± 80	0.299
After 6 months	170.9 ± 72	271.3 ± 86.6	< 0.0001*
Percent change between the 2 points	− 24.2 ± 15	11.1 ± 19	< 0.0001*
P Value within each arm	< 0.0001*	0.005*	
Serum creatinine (mg/dL)			
Baseline	1.1 ± 0.13	± 0.31.1	0.503
After 6 months	1.08 ± 0.09	1.2 ± 0.2	0.069*
Percent change between the 2 points	− 1 ± 8.2	5 ± 9.6	0.017*
P Value within each arm	0.245	0.014*	
eGFR (mL/min/1.73 m^2^)			
Baseline	84.7 ± 15	81.9 ± 18.7	0.528
After 6 months	85.4 ± 13.8	78 ± 14.1	0.040*
Percent change between the 2 points	1.5 ± 7.7	− 3.5 ± 8.8	0.029*
P Value within each arm	0.583	0.02*	

Values are expressed as mean ± SD

UACR, urinary albumin to creatinine ratio; eGFR, estimated Glomerular filtration rate

*Significant difference (P < 0.05)

### Secondary outcomes

After 6 months, a significant reduction in MMP-7 was revealed in the niclosamide arm—22% (95% CI, − 25.8 to − 18.2%), while there was a significant increase in the control arm. 16% (95% CI, 2.5 to 30%). Similarly, the niclosamide arm showed a significant decline in PCX after 6 months, while the control arm showed a significant rise. Niclosamide had no apparent effect on 8-OHdG as no statistically significant difference can be detected in both groups at baseline and the end of the study (Table 3).

**Table 3:** Summary of the secondary outcomes

	Niclosamide arm n = 30	Control arm n = 30	P value between arms
MMP-7 (ng/mL)			
Baseline	7.8 ± 1.4	7.4 ± 2	0.41
After 6 months	6.1 ± 1.1	8.1 ± 1.6	0.0001*
Percent change between the 2 points	− 22 ± 10	16 ± 36	< 0.0001*
P Value within each arm	< 0.0001*	0.003*	
PCX (ng/mL)			
Baseline	9.7 ± 5.2	9.5 ± 3.4	0.89
After 6 months	7.8 ± 3	10.9 ± 4.1	0.002*
Percent change between the 2 points	− 2 ± 53	15 ± 20	0.094
P Value within each arm	0.044*	0.001*	
8-OHdG (ng/mL)			
Baseline	34.3 ± 6.2	36.3 ± 7.3	0.27
After 6 months	38.4 ± 11.7	39.7 ± 9.8	0.66
Percent change between the 2 points	13 ± 36	12 ± 30	0.84
P Value within each arm	0.055	0.067	

Values are expressed as mean ± SD

*MMP-7* matrix metalloproteinase-7, *PCX* podocalyxin, *8-OHdG* 8-hydroxy-2ʹ-deoxyguanosine

*Significant difference (P < 0.05)

Fasting blood glucose didn’t differ markedly between the two arms after 6 months (165 mg/dL (± 20) and 173 mg/dL (± 28) for the niclosamide arm and the control arm respectively. However, hemoglobin A_1_C was significantly reduced in the niclosamide arm from 9.7 to 9.1% compared to the control arm that changed from 9.4 to 9.9% (P < 0.001).

Since hypertension and obesity may affect UACR, baseline values were investigated, and a sub-group analysis was performed. The mean UACR of the 22 obese patients was 232 mg/g (± 73) while the mean UACR of the 38 non-obese patients was 239 mg/g (± 87). No significant difference was revealed between the obese and non-obese patients with regards to the baseline UACR levels. Similarly, the baseline UACR of the 32 normotensive patients, was not statistically different from that of the 28 hypertensive patients (239 mg/g (± 80), vs 233 mg/g (± 84) respectively).

Sub-group analysis showed that niclosamide significantly reduce UACR in both normotensive patients and the hypertensive patients (− 27% (95% CI, − 44 to – 23%) vs -17% (95% CI, − 53 to − 22) respectively). Furthermore, niclosamide showed significant UACR reduction in both non-obese and obese patients (− 27% (95% CI, − 47 to − 28%) vs − 21% (95% CI, − 49 to − 10) respectively) (Table 4).

**Table 4:** Sub-group analysis of UACR based on hypertension or obesity

Hypertension	Niclosamide arm	Control arm	P value between arms
Percent change in normotensive	(n = 15)	(n = 17)	< 0.001*
	− 27% ± 6	17% ± 10	
Percent change in hypertensive	(n = 15)	(n = 13)	< 0.001*
	− 21% ± 12	17% ± 25	

Obesity	Niclosamide arm	Control arm	P value between arms
Percent change in non-obese	(n = 20)	(n = 18)	0.017*
	− 27% ± 16	10% ± 11	
Percent change in obese	(n = 10)	(n = 12)	< 0.001*
	− 17% ± 11	12% ± 27	

Values are expressed as mean ± SD

UACR, urinary albumin to creatinine ratio

*Significant difference (P < 0.05)

**Fig. 2:**
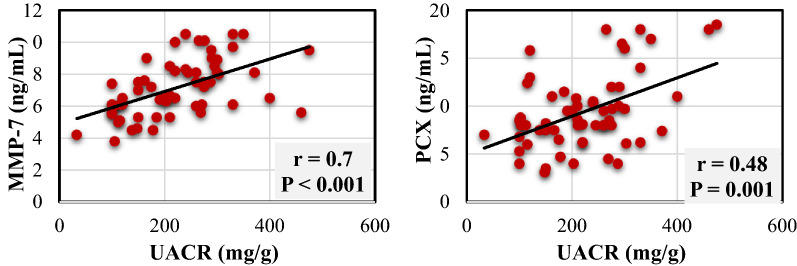
Correlations between UACR and both MMP-7 and PCX. *UACR* urinary albumin to creatinine ratio, *MMP-7* matrix metalloproteinase-7, *PCX* podocalyxin, *r* Pearson correlation coefficient

As presented in Fig. 2, correlation analysis showed a strong correlation between the change in UACR and the change in MMP-7 at the end of the study (r = 0.7, P < 0.001), while there was a moderate correlation between the change in UACR and change in the PCX at the end of the study (r = 0.48, P = 0.001).

Additionally, Among the changes in serum creatinine, eGFR, PCX, 8-OHdG, hemoglobin A1c, and fasting blood glucose, regression analysis revealed a strong association between the reduction in MMP-7 and the reduction in UACR. A 1 mg/dL decrease in MMP-7 level was associated with a 25 mg/g decrease in UACR (B = 24.95, P < 0.001).

Throughout the 6 months of the study, two patients in the niclosamide group complained of GIT upset. One of them was a 51 Y.O female who decided to discontinue niclosamide after taking it for 3 weeks and had declared that she had chronic dyspepsia before recruitment to the study and that taking that medication had exacerbated her condition. She withdrew from the study and was excluded from the analysis. The other patient was a 45 Y.O male who complained of mild GIT upset, he was advised, like other patients in the niclosamide arm, to take niclosamide just after meals and he continued taking niclosamide until the end of the study with no problems.

No other treatment-related side effect was reported. No recruited patients chronically use NSAIDS, depression medications, acetaminophen, antihistamines, or any other drugs known to induce albuminuria. However, some patients in both arms had been administered analgesics on an as-needed basis to treat a mild condition such as headache.

## Discussion

To our knowledge, this was the first randomized clinical trial to study the role of niclosamide on albuminuria in patients with DKD. This study demonstrated that niclosamide may reduce albuminuria in patients with type 2 diabetes mellites receiving ACEI. Patients in the niclosamide arm showed a significant decline in albumin excretion and were protected from the significant increase in albuminuria in the control arm. In addition, patients who received niclosamide preserved their serum creatinine and eGFR compared with the control arm.

Those outcomes suggest that niclosamide may be a promising therapeutic approach to protect against the progression of DKD and positively affect patients’ quality of life. These findings are consistent with previous preclinical trials that reported the ability of niclosamide to improve DKD in mice [13, 14]. Blocking Wnt/β-catenin signaling may be a possible mechanism behind the observed efficacy of niclosamide. Inhibiting mTOR signaling, decreasing podocyte injury, and improving beta cell function are additional mechanisms that may be involved in the niclosamide effect [13, 14].

Deterioration of albuminuria that occurred in the control arm despite using ACEI is in accordance with several studies that reported increase in albuminuria and progression of DKD despite using RAAS blocking agent [25–28].

Furthermore, MMP-7 was found to be elevated in all analyzed patients than normal reported urinary MMP-7 level of healthy volunteers confirming the fact that MMP-7 is elevated in diseased kidney [15, 16, 29]. Significant reduction of MMP-7 in the niclosamide arm, in addition to the strong association between MMP-7 and UACR, may increase the possibility that niclosamide may reduce albuminuria through inhibiting Wnt/β-catenin signaling.

The significant reduction in PCX level in the niclosamide arm, and the significant correlation between UACR and PCX level suggest that niclosamide may also protect against podocyte damage. Correspondingly, observations of a previous preclinical study reported niclosamide can recover podocyte dysfunction and ameliorate pathological renal injury [30]. Consistent with our correlation finding, two studies showed a good correlation between UACR and PCX [31, 32]. In contrast, one study showed a weak correlation between PCX and UACR [19]. Considering that the oxidative stress marker, 8-OHdG, was not affected by niclosamide use in the current study, it seems that niclosamide efficacy is not probably related to the reduction of oxidative stress.

The niclosamide arm showed slightly better glycemic control than the control arm which suggests that niclosamide may improve beta cell function. This finding is consistent with a previous preclinical study that reported the capability of niclosamide to improve pancreatic islet function, increase serum insulin levels and reverse hyperglycemia in mice with diabetes [13].

Niclosamide was reported recently to be a well-tolerated drug with no limiting toxicities even if it administrated in a dose of 2 g daily, which is a higher dose than used in this study [33]. Correspondingly, niclosamide was well tolerated and safely used in the present study with no major side effects reported except for some gastrointestinal upset experienced by only two patients.

This study has some potential limitations including small sample size, short duration, exclusion of patients with stage 4 and 5 chronic kidney disease, and high dropout rate.

## Conclusion

The present randomized clinical trial revealed that niclosamide may be effective as an adjuvant to ACEI in reducing albuminuria in patients with type 2 diabetes mellites. Blocking of Wnt/β-catenin signaling, decreasing podocyte injury, and improving Beta cell function are the suggested mechanisms underlying niclosamide efficacy. Large-scale clinical trials with longer duration are urgently needed for confirming our findings.

## Data Availability

The datasets used during the current study are available from the corresponding author on reasonable request.

## Abbreviations

8-OHdG: 8-Hydroxy-2ʹ-deoxyguanosine
ACEIs: Angiotensin-converting enzyme inhibitors
ARBs: Angiotensin receptor blockers
CKD-EPI: Chronic kidney disease epidemiological collaboration
DKD: Diabetic kidney disease
FDA: Food and Drug Administration
eGFR: Estimated glomerular filtration rate
ELISA: Enzyme-linked immunosorbent assay
ESRD: End-stage renal disease
MMP-7: Matrix metalloproteinase-7
mTOR: Mammalian target of rapamycin
PCX: Podocalyxin
RAAS: Renin–angiotensin–aldosterone system
UACR: Urinary albumin to creatinine ratio
